# A just-in-time huddle to prevent central line-associated bloodstream infections in high-risk children

**DOI:** 10.1017/ash.2025.10216

**Published:** 2025-12-10

**Authors:** Cristina Rios-Rivera, Lauren Edwards, Lindsey Garcia, Sarah B. Kandil, Thomas S. Murray

**Affiliations:** 1 Department of Pediatrics, Yale School of Medicine, New Haven, CT, USA; 2 Yale New Haven Children’s Hospital, New Haven, CT, USA

## Abstract

A central line-associated bloodstream infection prevention rapid response team (CLABSI-RRT) huddle was convened to provide just-in-time support to staff caring for patients with high-risk central venous catheters (CVCs). Two of the identified 46 high-risk children that had a huddle activated developed a CLABSI.

## Introduction

Central line-associated bloodstream infections (CLABSI) result in increased length of stay, costs, morbidity, and mortality.^1^ Examples of risk factors for CLABSIs in the pediatric population include prematurity, prolonged hospital stay, difficulty adhering to infection prevention techniques, and immunocompromise.^1^ Multiple interventions have been designed and assessed to reduce the incidence of CLABSI for neonates and children, including guidelines from the Society of Healthcare Epidemiologists of America and Children’s Hospitals’ Solutions for Patient Safety (SPS).^2,3^ Examples of these interventions include daily chlorohexidine gluconate baths, prevention bundles with monitoring of compliance, careful attention to central venous catheter (CVC) maintenance and dressing integrity, and daily assessment of the need for the CVC.^1–3^ Multidisciplinary teams have been an integral part of CLABSI reduction efforts.^4–6^ Teams convene on a regular basis to review CLABSI performance and compliance with ongoing prevention efforts or meet after a CLABSI to identify opportunities for practice improvement and to share information across multiple units.^5–7^ The purpose of this quality improvement (QI) initiative was to establish an early, formal mechanism to discuss the care of children with recognized risk for infection with the aim of reducing CLABSIs.

## Methods

Yale New Haven Children’s Hospital is a 212-bed tertiary care center in New Haven, Connecticut, with a 68-bed level IV neonatal intensive care unit and a 21-bed pediatric intensive care unit. In 2012, we introduced a CLABSI prevention bundle aligned with SPS, followed by a CLABSI QI task force to drive further performance improvement. This team includes representatives from front-line nursing, infection prevention, quality and safety, physician, and nursing leadership. Monthly meetings evaluate and revise the key driver diagram, review process measurement and outcome data, and perform case reviews. These reviews demonstrated the patient care team often recognized children with CLABSI risk factors (e.g., poor skin integrity, risk for external contamination) but was not aware of mitigation strategies. We utilized Plan Do Study Act (PDSA) cycles to address the key driver of recognizing children at increased CLABSI risk. Initial daily CVC assessments during clinical rounds were not routinely adopted because of time constraints.

**Figure 1.**
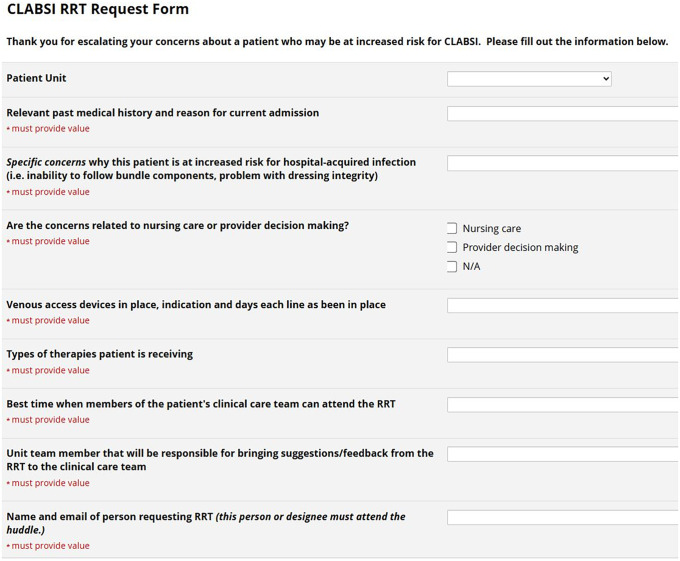
Standardized electronic information template to activate a CLABSI prevention Rapid Response Team huddle.

*The CLABSI prevention Rapid Response Team huddle.* Proactive safety huddles offer a strategy for adaptable and rapid risk mitigation in healthcare.^8^ Early identification of children with CVCs and risk factors for CLABSI by experts can reduce CLABSI rates.^9^ Given the inconsistency of daily assessments of children with CVCs during rounds, the next PDSA cycle created a formal proactive safety strategy for hospitalized children with a CVC; the just-in-time CLABSI prevention Rapid Response Team (CLABSI-RRT) huddle. Any patient care team member concerned about a patient’s CLABSI risk could email a Chair of the CLABSI QI Task Force to request a CLABSI-RRT huddle. Through PDSA cycles, we created a standard template for the huddle activation that included information about CLABSI risk (Figure 1). The Chair reviewed the request and confirmed it would benefit from the group’s expertise. Next, the CLABSI QI Task Force and unit leadership were invited via email to a virtual meeting scheduled within the next business day. CLABSI-RRT huddles routinely had 15–25 members, representing diverse nursing, physician, quality and safety, and infection prevention expertise. In 2024, an electronic form further standardized the information and automatically notified the CLABSI QI Task Force and unit leaders that a CLABSI-RRT huddle was activated (Figure 1).

Process measurement focused on how many CLABSI-RRT huddles were requested, the reason for the request, how many huddles took place, and if not, why a huddle did not occur. The primary outcome measure was whether patients who had a huddle activated developed a subsequent CLABSI.

## Results

From October 2020 to May 2025 there were 49 huddle requests, most commonly by nurses, for 46 patients with three patients receiving multiple requests (Table 1, Supplemental Figure 1). Fifteen huddles were not convened because a CLABSI QI Task Force leader helped resolve the issue by facilitating direct communication with the attending physician and patient care team (Table 1). This was the approach for all huddles activated within 24 hours of weekends and holidays, when the QI Task Force was not available. Additionally, the CLABSI-RRT huddle request often resulted in discussions about the CVC and action prior to a huddle convening. For example, two huddles were scheduled but canceled quickly as the CVC was removed. The most common reasons for a huddle were concerns about dressing integrity or skin breakdown (Table 1). From October 2020 to May 2025, our CLABSI rates varied from 1.17 to 1.45/1000 CVC days. The number of CLABSI-RRT huddles varied per quarter with a median of 1/month and an average of 2.5/month, S.D +/− 3.23. The number of proactive huddles trended up as CLABSI rates were increasing and there was an increased focus on CLABSI prevention (Supplementary Figure 1). One patient (2%) who had a CLABSI-RRT huddle activated and completed developed a CLABSI 27 days following the huddle, but with a new CVC in place. This older teenager had persistent difficulty with bundle adherence and environmental cleanliness. One patient (2%) had a CLABSI resulting from a blood culture drawn the night before the huddle was activated.

**Table 1.** Characteristics of CLABSI prevention Rapid Response Team huddles

Requested CLABSI Prevention Rapid Response Team (CLABSI-RRT) huddles (*N* = 49)	*N* (%)
*Reason for huddle*	
Dressing integrity Contamination Mechanical problems Skin integrity CVC necessity Discomfort with catheter type Two of the above reasons	23 (40) 11 (19) 9 (16) 8 (14) 5 (9) 1 (2) 8 (14)
*Huddle completed within 24 hours of request*	34 (69)
*Reasons a huddle was declined*	15 (45)
CVC removed/exchanged prior to RRT Escalation up the chain of command Care plan put in place before huddle Patient Relations/Unit leadership involvement	6 (40) 5 (33) 3 (20) 1 (66)
*CVC removed within 24 hours of huddle*	9 (18)
*CVCs with CLABSI after huddle*	0 (0)
Patients with CLABSI during admission*	2 (4)
*Unit requesting huddle*	49 (100)
Neonatal Intensive Care Pediatric Intensive Care Pediatric Cardiac Intensive Care General Medical/Surgical Hematology/Oncology	16 (33) 14 (29) 7 (14) 7 (14) 5 (10)
*Role requesting huddle*	
RN MD MD, RN RN, APRN	44 (90) 3 (6) 1 (2) 1 (2)

CLABSI, Central line associated bloodstream infection; CVC, central venous catheter; RRT, Rapid Response Team

* One CLABSI was a result of a blood culture drawn the night before the huddle was completed. The other was a patient with a different CVC than the one present during the huddle. The CLABSI occurred 27 days after the initial CLABSI-RRT huddle.

## Discussion

To our knowledge this is the first description of a hospital-wide just-in-time proactive safety huddle to discuss strategies to prevent CLABSI for children perceived to be at high risk. Proactive huddles reduced CLABSIs in a hematology/oncology population as part of a larger QI initiative.^6^ Krauss et al identified themes for CLABSI prevention that included facilitation of idea sharing, preexisting infrastructure, and broad buy-in.^10^ The just-in-time CLABSI RRT huddle proactively accomplishes these goals. An expert, multidisciplinary team offered individualized strategies to reduce CLABSI risks. When provided with a formal mechanism, staff felt empowered to escalate concerns about CVCs. We achieved broad buy-in with consistent representation from multiple units and disciplines for all huddles. The CLABSI-RRT huddles have many of the core attributes of safety huddles that successfully mitigate risk in healthcare.^8^ They were multidisciplinary and fostered communication, had sustained support from the CLABSI QI Task Force and unit leadership, and were time bound and goal oriented.^8^

Through these huddles, we also identified opportunities for system improvement. For example, barrier mitigation strategies such as a plastic drape or “mud flap” initially used in the intensive care unit to prevent external contamination spread to other inpatient units. These huddles also helped keep CLABSI prevention at the forefront of daily clinical care. Current work emphasizes the availability of these huddles when CLABSI rates are lower. We hypothesize that learned strategies as a result of the CLABSI-RRT huddles were applied to other patients with CVCs, improving care without the need to huddle.

## Limitations

There are limitations that make it difficult to fully understand the value of this intervention. Due to the multifactorial reasons for CLABSIs and multiple QI initiatives, our CLABSI rates remained highly variable during the implementation period (Supplemental Figure 1). We did not track which interventions were adopted because the huddle often resulted in multiple suggestions for the primary team. We did not huddle within 24 hours of a request on weekends and holidays because logistically QI CLABSI Task Force members were not available. Instead, assistance was provided by CLABSI QI Task Force leadership.

## Conclusion

Proactive CLABSI-RRT huddles for children perceived to be high risk for infection increased awareness of CLABSI mitigation strategies, spread best practices, and promoted interdisciplinary communication.

## Supporting information

10.1017/ash.2025.10216.sm001 Rios-Rivera et al. supplementary material Rios-Rivera et al. supplementary material

## Supplementary material

The supplementary material for this article can be found at https://doi.org/10.1017/ash.2025.10216.
