# Supplementary figures and images for: A just-in-time huddle to prevent central line-associated bloodstream infections in high-risk children
Source: Antimicrob Steward Healthc Epidemiol. 2025 Dec 10;5(1):e331. doi: 10.1017/ash.2025.10216 (PMC12722551; doi:10.1017/ash.2025.10216)

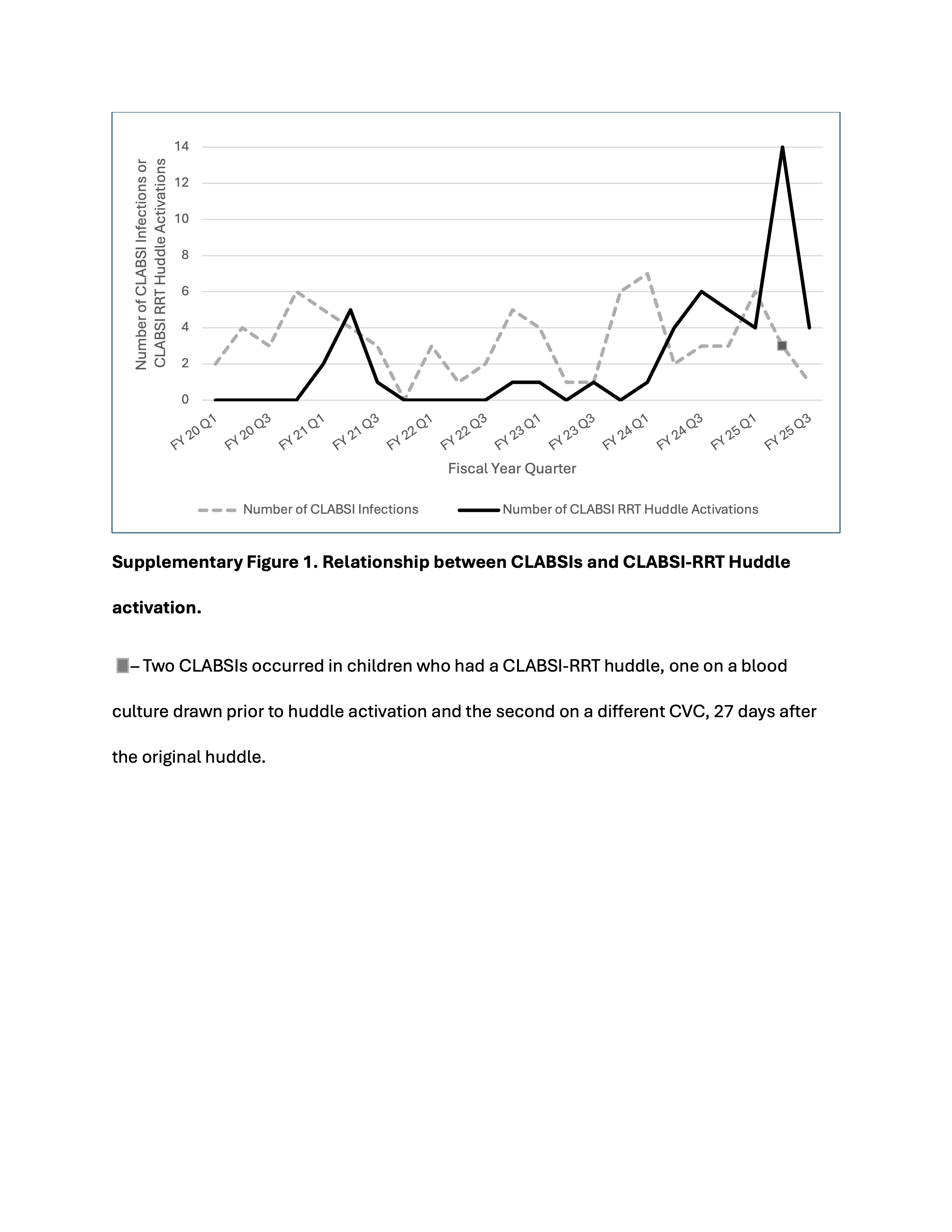

Supplement: Rios-Rivera et al. supplementary material [file S2732494X25102167sup001.tiff]
